# Characterization of *Mycobacterium salfingeri* sp. nov.: A novel nontuberculous mycobacteria isolated from a human wound infection

**DOI:** 10.3389/fmicb.2022.992610

**Published:** 2022-10-10

**Authors:** Emily Musser, Carol Smith, Tanya A. Halse, Donna Kohlerschmidt, Amy Rourke, Alexandra Fiero, Kimberlee A. Musser, Vincent Escuyer, Pascal Lapierre

**Affiliations:** Wadsworth Center, New York State Department of Health, Albany, NY, United States

**Keywords:** nontuberculous mycobacteria, novel species, *Mycobacterium salfingeri*, human pathogen, genome

## Abstract

Nontuberculous mycobacteria (NTM) are environmental bacteria commonly found in soil and water in almost every part of the world. While usually non-pathogenic, they can cause acute respiratory and cutaneous infections under certain circumstances or in patients with underlying medical conditions. Contrary to members of the *Mycobacterium tuberculosis* complex, documented human-to-human transmissions of NTM have been rarely reported and most cases result from direct environmental exposure. Here we describe the identification of a new NTM species isolated from a hand laceration of a New York State patient after a fall. This new NTM forms rough, orange pigmented colonies and is naturally resistant to doxycycline and tobramycin. Whole genome analysis reveal no close relatives present in public databases, and our findings are in accordance with the recognition of a new taxonomic species of NTM. We propose the name *Mycobacterium salfingeri* sp. nov. for this new NTM representative. The type strain is 20-157661T (DSM = 113368T, BCCM = ITM 501207T).

## Introduction

Nontuberculous mycobacteria (NTM) are ubiquitous, free-living environmental bacteria that do not cause tuberculosis or leprosy (Forbes et al., 2018; Johansen et al., 2020). They form a very diverse group of organisms in terms of geographic distribution, phylogeny, genome sizes, plasmid content, and are often classified into two distinct groups of slowly and rapidly growing mycobacteria (Hoefsloot et al., 2013; Johnson and Odell, 2014; Fedrizzi et al., 2017; Tortoli et al., 2017; Turenne, 2019). NTMs are mostly non-pathogenic to humans except under certain circumstances or in association with underlying medical conditions (Cook, 2010; Ratnatunga et al., 2020). In the past, they were often dismissed as medically unimportant, but recent rises in cases involving NTMs worldwide have shifted the view of this group as pathogens of concern (Johnson and Odell, 2014; Donohue, 2018; Ratnatunga et al., 2020). The most clinically significant of the slowly growing NTM species include *Mycobacterium avium*, *M. intracellulare*, *M. kansasii*, *M. marinum*, *M. xenopi*, *M. malmoense*, *M. haemophilum*, and *M*. *ulcerans* (Caulfield et al., 2019); the common rapidly growing species to cause clinical disease are *M. abscessus*, *M. fortuitum* group and *M. chelonae*, and its subspecies (Brown-Elliot and Wallace, 2019). Nontuberculous mycobacteria are frequently resistant to multiple drugs, leading to prolonged and costly treatments (van Ingen et al., 2012), and a pressing need for better characterization and understanding of the diversity among NTMs. In 2018, Gupta et al. proposed the classification of NTMs into four newly created genera within the genus *Mycobacterium* (*Mycolicibacterium, Mycolicibacillus, Mycolicibacter* and *Mycobacteroides*) based on shared molecular markers specific to each genera (Gupta et al., 2018). Another study looking at the evolutionary relationship of the proposed new genera concluded that a unified *Mycobacterium* genus should be favored over the five genera split (Meehan et al., 2021). Confusion among researchers and clinicians regarding this new proposed nomenclature has led to the accepted coexistence of the traditional use of the *Mycobacterium* genus and the new nomenclature for naming NTMs (Tortoli et al., 2019).

Here we present the case of a 19-year-old New York State resident who was evaluated for a wound infection in her left hand. She stated that she had fallen on the ground 3 days earlier and presented with a laceration in the palm of her hand. The treating facility was able to grow *Streptococcus mitis* and an anaerobic gram-negative rod from the wound, but the infection worsened despite administration of a standard antibacterial treatment with amoxicillin/clavulanate potassium (augmentin), and another acid-fast isolate cultured on Löwenstein–Jensen medium (LJ), was sent to the Wadsworth Center for further evaluation. This isolate (internally named strain 20-157661T), was not readily identified by our typical testing algorithms, including real-time PCR, matrix-assisted laser desorption ionization-time of flight mass spectrometry (MALDI-TOF MS), and Sanger gene sequencing of *rpoB*, *hsp65*,16S rRNA and Internal Transcribed Spacer (ITS) targets. As a result, we used whole genome sequencing (WGS) to assist with identification. Database comparisons of 16S rRNA and other gene sequences and whole-genome phylogeny were unable to identify a known species of NTM as the causative agent of this infection.

## Materials and methods

Our laboratory received an acid-fast isolate cultured on Löwenstein–Jensen medium (LJ) from the treating facility. Sample processing and an in-house-developed real-time PCR assay specific to the *Mycobacterium tuberculosis* complex (MTBC) and *Mycobacterium avium* complex (MAC) were initially performed as previously described in Halse et al. (2010); Tran et al. (2014). An additional real-time PCR targeting *erm* to identify *M. abscessus* was also performed (Zhu et al., 2015). Matrix-Assisted Laser Desorption Ionization-Time of Flight Mass Spectrometry (MALDI-TOF MS; Biotyper; Bruker Daltonics, Billerica, MA, United States) was performed for species-level identification (Rodriguez-Temporal et al., 2020). Our laboratory utilizes a 1.8 cutoff score for identification. Amplification of a 750-bp portion of the *rpoB* and 450-bp region of the *hsp65* genes was performed utilizing oligonucleotide primers specific to known conserved regions that flank the most variable regions of the genes (Adékambi et al., 2003; McNabb et al., 2004). Additionally, a 500-bp portion of the 16S rRNA gene (Lane et al., 1985; Weisburg et al., 1991) and a 473-bp region of the ITS region were amplified (Frothingham and Wilson, 1993). Culture samples were prepared for Sanger sequencing by cleaning PCR products with ExoSAP-IT (Thermo Fisher Scientific, Waltham, MA, United States) to remove unwanted dNTPs and primers. DNA sequences were compared to sequences found in National Center for Biotechnology Information (NCBI) using BLAST, the MicroSEQ™ ID Analysis Software, and the EZTaxon database (for 16S rDNA analysis only)^1^ to determine the identification.

Subculture was performed by incubation at 37°C without CO_2_ on both Middlebrook 7H10 and LJ medium and incubated for up to 8 weeks. Colony morphology was recorded after 10 days and additional incubation time at 30°C and 42°C was performed with and without light exposure. Microscopic examination, which included Ziehl-Neelsen acid-fast staining, confirmed the presence of acid-fast bacilli (AFB) and was utilized to describe the size, formation, and other characteristics. Biochemical characterization was performed as previously described by Kent and Kubica (1985) except for the β-glucosidase that was tested by following the procedure described by David and Jahan (1977). Antimicrobial susceptibility testing was performed by broth microdilution assay, using commercially available microplates (RAPMYCO2; Thermo-Fisher) and following the Clinical and Laboratory Standards Institute (CLSI) guidelines (M24Ed3 | Susceptibility Testing of Mycobacteria, Nocardia spp., and Other Aerobic Actinomycetes, 3rd Edition, 2018). Antimicrobial agents tested include amikacin, cefoxitin, ciprofloxacin, clarithromycin, clofazimine, doxycycline, imipenem, linezolid, moxifloxacin, tigecycline, tobramycin and trimethoprim/sulfamethoxazole. A database search to identify the presence on antimicrobial resistance genes in the genome was also performed using Abricate^2^ and ResFinder (Florensa et al., 2022).

For whole genome sequencing (WGS), DNA was extracted from 1 ml of heat-inactivated liquid MGIT culture using a modified version of the InstaGene/FastPrep (IG/FP) method described by Shea et al. (2017). Specifically, the 56°C incubation was reduced from 30 to 10 min, and the volume of InstaGene matrix added to the pellets was altered to be 130–200 μl, based on the size of the pellet observed. These changes were implemented to reduce extraction turnaround time and increase DNA yield. DNA yields were measured by Qubit fluorometry. Illumina MiSeq WGS was performed using Nextera XT sequencing library, paired-end 250-bp with 15 PCR cycles for the indexing step. DNA extraction for long-read sequencing was performed following the protocol described for procedure #1 in Epperson and Strong (2020) with some modifications. Briefly, cells were resuspended in lysis buffer by gentle pipetting. Following the addition of lysozyme, cells were incubated for 2 h at 37°C with gentle rotation. Proteinase K and SDS were added to the cells and were incubated at 50°C for 30 min. Genomic DNA was extracted using the Genomic DNA Clean and Concentrator Kit (Zymo Research). ChIP Binding Buffer was added, and the mixture was inverted gently to mix. The sample was spun at 4,500*g* for 3 min, and the supernatant was applied to the column. The column was washed twice with wash buffer, and two elutions were performed. The first elution was in 10 mM Tris, pH 8.0. This was added to the column and, after a 5 min incubation at room temperature (18°C–28°C), the column was spun to elute the genomic DNA. The eluate was applied back onto the same column, allowed to incubate for 5 min at room temperature, and then spun for the second elution. Nanopore sequencing was performed on the Oxford Nanopore MinION Mk1C platform using the SQK-LSK109 ligation sequencing kit according to the manufacturer’s instructions on a FLO-MIN106 flow cell. The reads were basecalled with Guppy v.4.2.3 using the Fast Basecalling model. Genome assembly was performed with Flye version 2.8.3-b1695 (Kolmogorov et al., 2019) using the nanopore reads only, and MiSeq reads were used for two rounds of polishing using Nextpolish v1.3.1 (Hu et al., 2020). Final genome annotation was performed with the NCBI Prokaryotic Genome Annotation Pipeline (PGAP) 2021-01-11.build5132 (Tatusova et al., 2016).

A dataset of 26 related *Mycobacterium* species/subspecies with complete genomes was compiled from NCBI with BLAST searches using multiple random 25 kilobases portion of the strain 20-157661T genome assembly as query. C*orynebacterium pseudotuberculosis* PA09 was selected to serve as a distantly related genus for genome comparisons and phylogenetic analyses. Average nucleotide identity (ANI) between each pair of genomes was determined using FastANI (Jain et al., 2018). 16S rRNA retrieved from the same 26 related type strain NTM genomes (Supplementary Table 1) was used to assess the phylogenetic relationships of the NTM in our dataset using MAFFT (Katoh et al., 2002) to align the sequences and IQ-TREE (Nguyen et al., 2015) with 1,000 bootstraps and auto model selection to calculate a maximum likelihood tree. OrthoFinder (Emms and Kelly, 2017, 2018, 2019) with default parameters was used to extract single-copy orthologous core genes and build a species tree from the concatenated core protein sequences. Calculation of the percent identity matrix from the MAFFT aligned sequenced included both nucleotide and indels. Pan-genome analyses were performed using Roary version 3.13.0 (Page et al., 2015), using 85% protein identity cutoff for homologous sequences on the re-annotated genomes with Prokka version 1.14.5 (Seemann, 2014) without plasmid sequences included.

## Results and discussion

The number of NTM cases yearly diagnosed worldwide has been steadily increasing over the past two decades (Prevots and Marras, 2015; Ratnatunga et al., 2020). While the cause for this increase is still not fully understood, a better understanding of NTM and increased availability of diagnostic tools are likely partially responsible. Yet, our extent of knowledge on the prevalence and diversity of NTM in the environment is still lacking. As such, it is important to continue to study and characterize NTM to increase our comprehension and testing available to accurately diagnose NTM infections to better inform patient treatment and lower the burden of those infections.

This case originates from a young adult, resident of New York State, who sought medical care for an infected hand wound after a fall to the ground. Initial treatments failed to resolve the infection, and subsequent attempts to identify the cause of the infection by real-time PCR were unsuccessful, providing evidence of a new NTM species. Clinical outcome or risk factor information on the patient have not become available. Several studies have shown that MALDI-TOF MS can reliably be used to identify NTM at the species level in more than 95% of the cases when using solid media culture (Mediavilla-Gradolph et al., 2015; Genc et al., 2018; Rodriguez-Temporal et al., 2020). MALDI-TOF MS identifications are determined using log (score) ranging from 0 to 3, derived by comparing the peak profile of the query sample against a database of main spectrum profiles from known species. Our attempt to identify this isolate using MALDI-TOF MS was unsuccessful, and the closest recovered match was *M. chitae* with a score of 1.39, well below the standard score cutoff for species identification of 1.7 as determined by the manufacturer, and our approved NY protocol utilizing a score of 1.8. Similarly, database searches using targeted Sanger sequencing resulted in best BLAST matches to *Mycobacterium rutilum* for *rpoB* gene (93.5% identity), *Mycobacterium novocastrense* for *hsp65* gene (93.1% identity), *Mycobacterium moriokaense* for 16S rRNA gene (94.6% identity), and *Mycobacterium gadium* for the ITS ribosomal RNA (86.0% identity), which did not meet our criteria for identification.

The genetic determinants for NTM growth phenotype, i.e., slow versus fast growers, are still unknown. By convention, 6 days to form visible colonies on solid media is the cutoff to determine if a species is a slow or fast grower. In this presented case, strain 20-157661T took a total of 10 days to form visible colonies on both Middlebrook 7H10 and LJ medium at 37°C without CO_2_, which is relatively fast for a slow grower. Nevertheless, while phylogenetically closer to other fast growers, based on the accepted nomenclature, it is still considered a slow grower.

**Figure 1:**
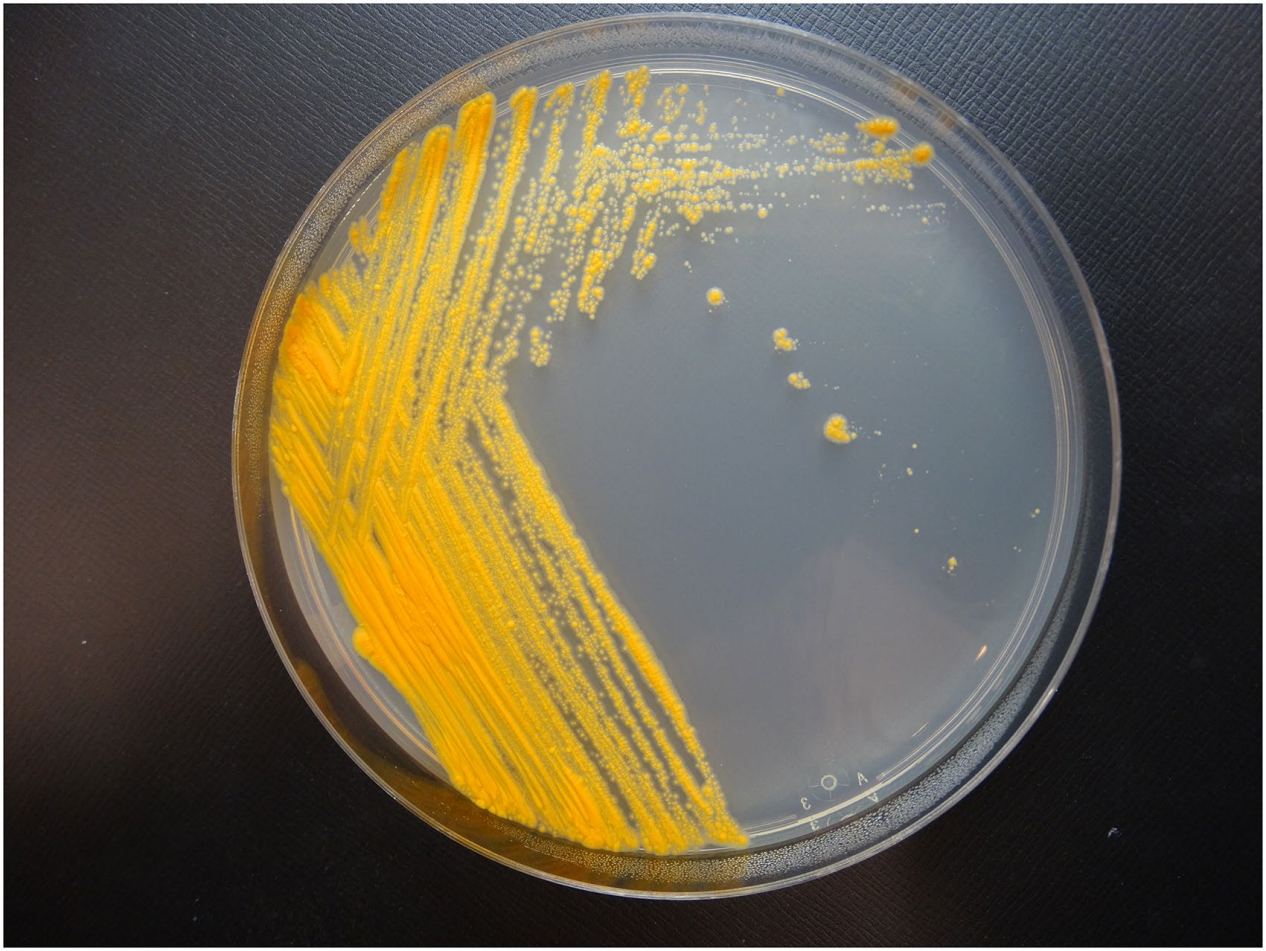
Colony morphology of strain 20-157661T after 2  weeks incubation at 37°C on Middlebrook 7H10 agar plate.

Colony morphology on both medias were round, dry, raised with irregular margins, rough in appearance, and orange in color (Figure 1). After 7 weeks of incubation, the colonies became vibrant orange with prominent pulvinate centers and varying widths of raised irregular margins and dry in appearance. The colonies were scotochromogenic, i.e., the orange pigmentation was produced regardless of light exposure or incubation temperature (Figure 1). The isolate grew well at 30°C and 37°C but no growth was observed at 42°C. Microscopic examination after Ziehl-Neelsen acid-fast staining confirmed the presence of AFB that was 1–3 μm in length in dot form clusters (Tu et al., 2003) and beading without cording. Biochemical characterization was performed, and the results are summarized in Table 1. More notably, strain 20-157661T was found to be negative for production of niacin and nitrate. We did not detect a thermostable catalase activity, could not reduce tellurite but iron uptake was detected. Strain 20-157661T was able to grow on mannitol as carbon source, but not citrate or inositol. Antimicrobial susceptibility testing determined the strain was resistant to doxycycline and tobramycin, intermediately susceptible to imipenem, and susceptible to all other agents.

**Table 1:** Phenotypic characteristics of strain 20-157661T.

Phenotypic characters	20-157661T	*Mycobacterium moriokaense* ^a^ ^,^ ^b^	*Mycobacterium pulveris* ^b^	*Mycobacterium phlei* ^c^ ^,^ ^d^	*Mycobacterium celeriflavum* ^e^
Pigment Production	Yes	No	No	Yes	Yes
Pigment Classification	Scotochromogen	Nonphotochromogen	Nonphotochromogen	Scotochromogen	Scotochromogen
Pigment Produced 30°C	Orange	No pigments	No pigments	Yellow/Orange	Yellow
Pigment Produced 37°C	Orange	No pigments	No pigments	Yellow/Orange	Yellow
Growth rate	Slow	Rapid	Rapid	Rapid	Rapid
Colony Morphology	Rough	Rough	Smooth	Rough	Rough
Growth at 42°C	No growth	Growth Present	Growth Present	Growth Present	No growth
Arylsulfatase (14 Days)	+	+	+	+	ND
Niacin	−	−	−	−	−
Nitrate	−	+	+	+	+
Tween 80 Hydrolysis	+ On Day 5	+	+	+	−
Urease	+ On Day 3	+	+	+	Variable
β-glucosidase	−	−	−	−	−
Thermostable Catalase	−	−	+	+	+
Semiquantitative Catalase	−	−	+	+	Variable
Lowenstein-Jensen w/5% NaCl	Growth present	Growth present	Growth present	Growth present	Growth present
MacConkey w/out Crystal Violet	No growth	ND	ND	ND	No growth
Tellurite Reduction	−	ND	ND	ND	+
Iron Uptake	+	ND	ND	ND	ND
**Carbohydrate utilization**					
Citrate	No growth	No growth	No growth	Growth present	ND
Mannitol	Growth present	Growth present	No growth	Growth present	ND
Inositol	No growth	Growth present	No growth	No growth	ND

ND, Not Determined.

a Tsukamura et al. (1986).

b Tsukamura and Ichiyama (1986).

c Bahram et al. (2012).

d Tsukamura et al. (1968).

e Shahraki et al. (2015)

WGS and assembly resulted in the recovery of two complete circularized DNA fragments; a main chromosome of 4,980,651 base pair and a 191,242 base pair plasmid, ranking strain 20-157661T as one of the smaller genomes among fully sequenced NTM (Table 2). The accuracy and completeness of the genome assembly was determined using Busco v5.4.3 (Simão et al., 2015). A total of 99.6% of the expected gene set (740/743 genes) was complete, with only 1 gene that was found to be fragmented and 2 missing genes. Genome annotations predicted a total of 5,027 coding sequences (CDS) with two copies of ribosomal RNAs (5S, 16S, 23S) and 46 tRNAs. The presence of a plasmid in strain 20-157661T is not unusual as about a third of the NTM genomes in this analysis contains between one and three plasmids. BLAST searches of this plasmid sequence only resulted in partial matches against the non-redundant database with a best match belonging to *Mycobacterium* sp. PYR15 with 97.1% identity over 45% of coverage. This plasmid encodes, among other things, a type-VII secretion system operon, mammalian cell entry (MCE) protein family, and type II toxin-antitoxin systems. These protein systems are most likely involved in the defense mechanisms of NTMs to help protect or evade potential threats, which indirectly include the human immune system (Zaychikova et al., 2015; Rivera-Calzada et al., 2021; Klepp et al., 2022). A type b aminoglycoside-modifying enzyme (aac(2′)-Ib) and a tetracycline efflux MFS transporter Tet(V) was also present on the main chromosome which would correlate with the natural resistance of this strain to tobramycin and doxycycline.

**Table 2:** Genome sizes, plasmid numbers, predicted coding proteins, rRNAs and tRNAs.

	Genome size (Mb)	Plasmid(s)	Proteins	rRNA	tRNA
*Mycobacterium doricum* JCM 12405	4.03	0	3,974	6	51
*Mycobacterium tuberculosis* H37Rv	4.41	0	3,906	3	45
*Mycobacterium celeriflavum* JCM 18439	5.02	0	4,865	6	51
**20-157661T**	**5.17**	**1**	**5,027**	**6**	**46**
*Mycobacterium phlei* CCUG 21000	5.35	0	5,149	6	59
*Mycobacterium avium* 104	5.48	0	5,120	3	46
*Mycobacterium pulveris* JCM 6370	5.48	0	5,376	6	50
*Mycobacterium litorale* JCM 17423	5.58	0	5,387	6	60
*Mycobacterium avium* subsp*. hominissuis* JP-H-1	5.73	3	5,469	3	55
*Mycobacterium phocaicum* JCM 15301	5.85	0	5,691	6	63
*Mycobacterium madagascariense* JCM 13574	5.94	1	5,690	6	53
*Mycobacterium gadium* JCM 12688	5.96	0	5,917	6	47
*Mycobacterium branderi* JCM 12687	5.98	1	5,860	6	51
*Mycobacterium monacense* JCM 15658	6.04	0	5,872	6	56
*Mycobacterium mantenii* JCM 18113	6.19	0	5,839	3	53
*Mycobacterium stomatepiae* JCM 17783	6.21	0	6,109	3	56
*Mycobacterium florentinum* JCM 14740	6.23	0	5,914	3	55
*Mycobacterium moriokaense* JCM 6375	6.23	0	6,133	6	48
*Mycobacterium vaccae* 95051	6.24	0	5,722	6	48
*Mycobacterium gallinarum* JCM 6399	6.30	1	6,202	6	49
*Mycobacterium chubuense* NBB4	6.34	2	5,843	6	46
*Mycobacterium rhodesiae* NBB3	6.42	0	6,147	6	46
*Mycobacterium arabiense* JCM 18538	6.45	1	6,179	6	53
*Mycobacterium vanbaalenii* PYR-1	6.49	0	5,979	6	49
*Mycobacterium smegmatis* MC2 155	6.99	0	6,452	6	47
*Mycobacterium goodii* X7B	7.11	0	6,321	6	47
*Mycobacterium mageritense* JCM 12375	8.01	0	7,831	6	93

**Figure 2:**
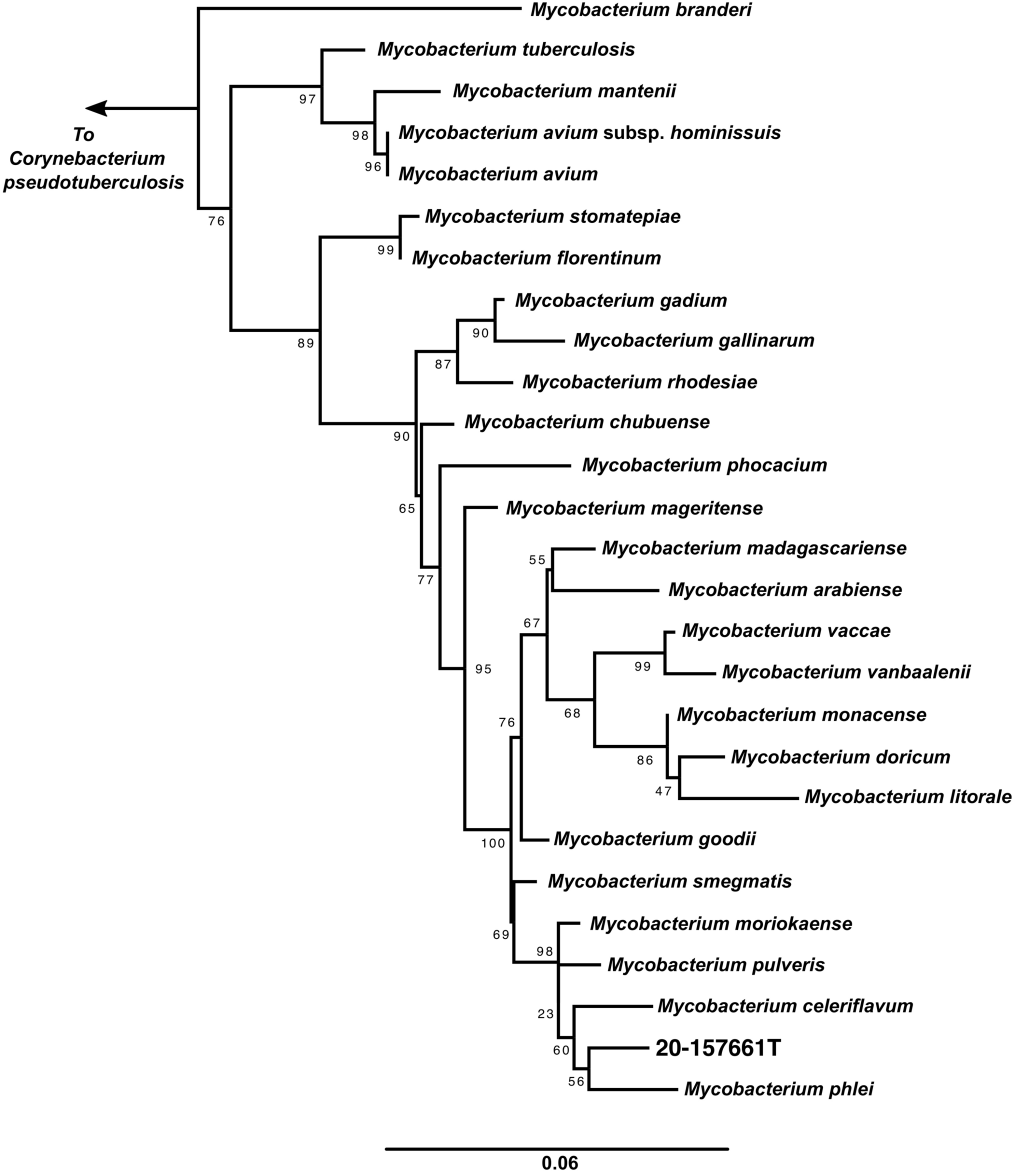
16S rRNA maximum likelihood tree. 16S rRNA sequences from the representative genomes in the dataset were aligned using MAFFT (Katoh et al., 2002) and IQ-TREE (Nguyen et al., 2015) was used to calculate the tree with 1,000 bootstraps replicates and using the auto model selection. *Corynebacterium pseudotuberculosis* was used to root the tree.

A maximum likelihood tree of the aligned full-length 16S rRNA sequences was calculated to assess the placement of strain 20-157661T in a phylogenetic context (Figure 2). While at the individual level, the bootstrap supports were too low to ascertain which species is the closest relative to strain 20-157661T, there were strong statistical support indicating that this strain clustered with a clade composed of *M. phlei, M. pulveris, M. celeriflavum* and *M. moriokaense*. It is commonly accepted that in most cases, full-length 16S RNA sequences with percent identity below 98.5% is enough to delineate between different bacterial species (Rodriguez-R et al., 2018), although other studies propose a 98.65% cutoff for species definition (Kim et al., 2014). In the case of strain 20-157661T, all bacterial species except for one, have a percent identity on the 16S level of 98.31% or lower, showing that they are indeed different mycobacterial species compared with strain 20-157661T (Supplementary Figure 1). The only exception is with *M. moriokaense* with 98.57% identity which according to the 98.65% cutoff, will classify the strain as a new species but not under the 98.5% cutoff. However, revised studies of 16S sequence identities across different bacterial genera, including the *Mycobacterium* genus, have shown that multiple clades do not conform to established identity cutoffs and that either higher cutoffs or other classification methods should be applied (Beye et al., 2017; Rodriguez-R et al., 2018).

One such method calculates the ANI across each pairwise genome comparison (Rodriguez-R and Konstantinidis, 2014). This method, akin to the *in vitro* DNA–DNA hybridization technique (Wayne et al., 1987), uses pairwise assembled genome alignments to determine how similar or dissimilar two samples are. In general, a ~95% to 96% ANI cutoff is recommended for species delineation with at least 80% of both genomes aligned (Richter and Rosselló-Móra, 2009; Chun et al., 2018; Ciufo et al., 2018). When comparing this isolate against our dataset on NTM genomes, the highest ANI score obtained belongs to *M. celeriflavum* with 82.83% average nucleotide identity, well below the 95% species cutoff (Supplementary Figure 2). This demonstrates that although the 16S rRNA comparisons were ambiguous, under ANI definitions, this isolate should be classified as a new species in the *Mycobacterium* genus.

**Figure 3:**
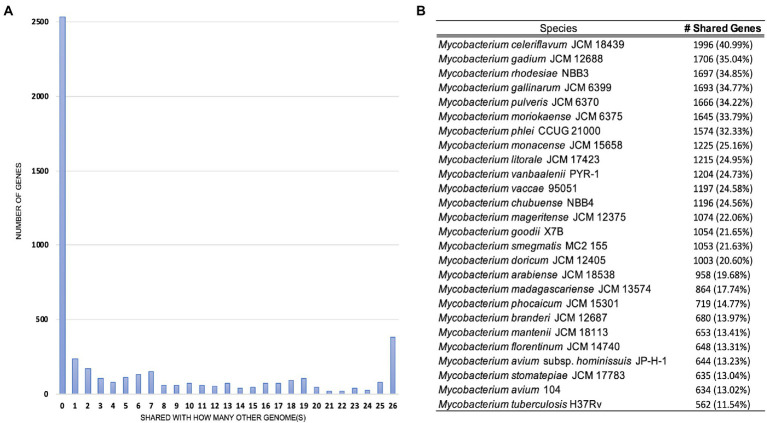
**(A)** Number of genomes in which genes in strain 20-157661T have homologous sequences. The first bar on the left-hand side represents genes unique to strain 20-157661T (48%) while the last bar on the right represents the core genomes, i.e., genes present in all the analyzed genomes (7.8%). **(B)** Number of homologous strain 20-157661T genes shared by each of the genomes in our dataset. Homologous genes were defined as being at least 85% identical over their aligned protein sequences.

**Figure 4:**
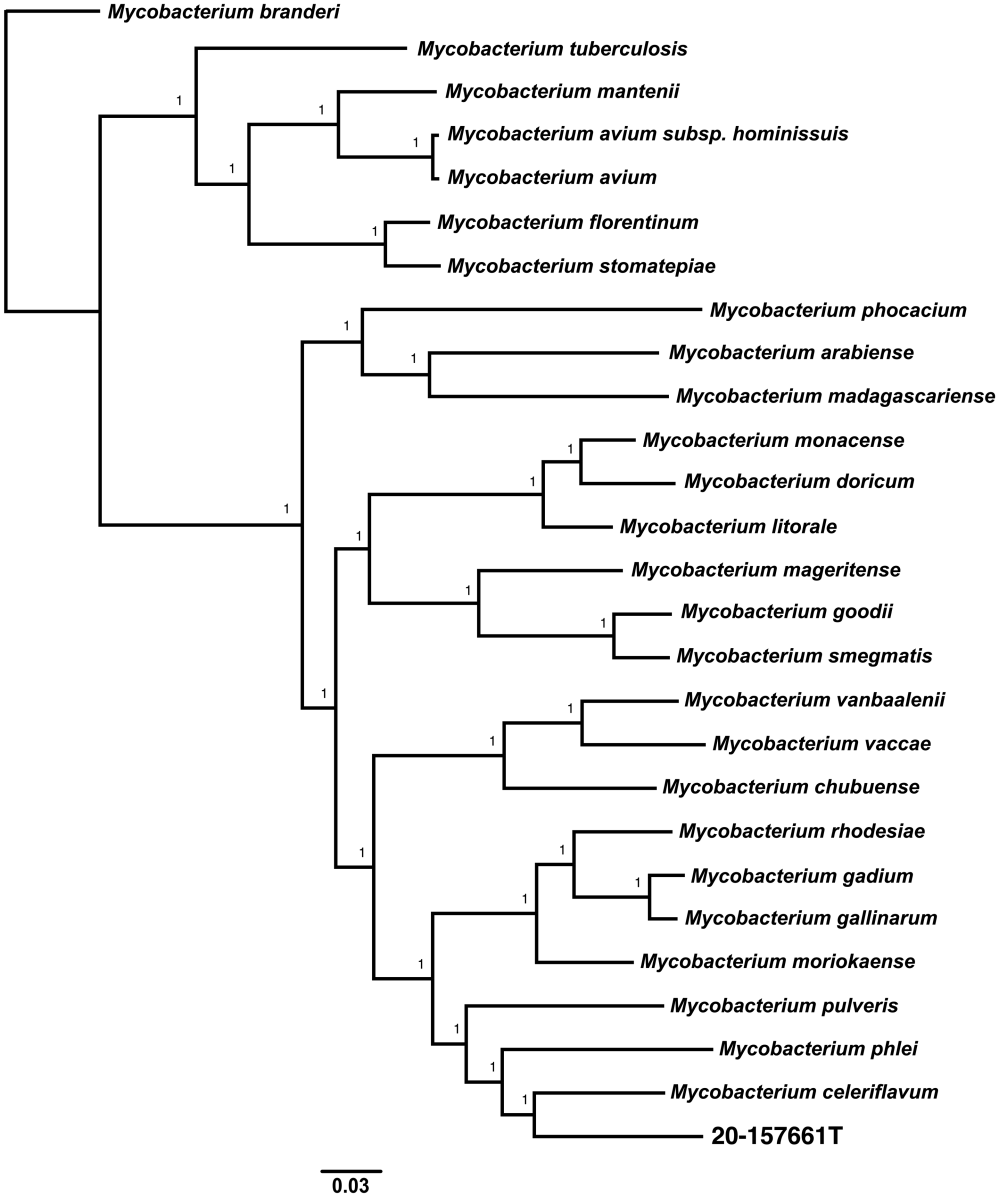
Unrooted core genome species tree calculated using 1,083 concatenated single copies orthologous protein sequences. The core genome alignment was generated using OrthoFinder v2.5.4 (Emms and Kelly, 2017, 2018, 2019) using default parameters. The scale bar represents the number of amino-acids substitutions per site. A Shimodaira-Hasegawa (SH) test was used to calculate the statistical support of the tree branches.

Pan-genome analyses using an 85% protein identity cutoff revealed that of the 4,869 predicted chromosomal genes by Prokka, 2,533 (48%) genes were unique to this isolate when compared to the 26 other genomes in our dataset (Figure 3A). A total of 380 (7.8%) genes were shared among all the genomes forming the core genome of our dataset, with the remainder of the genes being shared with at least one other species. At the individual genome level, *M. celeriflavum* was the species that had the most homologous genes in common with only 1,996 (41%) of the genes in strain 20-157661T shared between the two genomes (Figure 3B). Species tree calculated using a concatenation of 1,083 core proteins identified with OrthoFinder confirms the placement of strain 20-157661 as a sister relative to *M. celeriflavum* with strong Shimodaira-Hasegawa support values (Figure 4). The pan-genome analyses of strain 20-157661T also offer compelling evidence that this strain is unique with many genomic features not found in any of the other species. Close to 50% of its genes do not have homologous sequences in any of the other genomes, and only 41% are shared with its closest relative in our dataset (*M. celeriflavum*). Its 191,242 base pair plasmid also appears to be fairly unique, not only within the *Mycobacterium* genus, but also within the NR database where only partial BLAST hits could be recovered. For these reasons, we propose this newly characterized isolate, *M. salfingeri* sp. nov, as a new species within the *Mycobacterium* genus.

### Description of *Mycobacterium salfingeri* sp. nov

We propose the name *Mycobacterium salfingeri* sp. nov [sal.fin’ge.ri N.L. gen. n. salfingeri (italics)] for this new species for the pioneering contributions of Dr. Max Salfinger, a Swiss-born American microbiologist in the field of clinical mycobacteriology. *Mycobacterium salfingeri* sp. nov cells are bacilli-shaped, acid-fast, 1–3 μm in length in dot form clusters. This slowly growing NTM strain forms vibrant orange colonies after incubation at 37°C for 2 weeks with prominent pulvinate centers and varying width of raised irregular margins and a dry in appearance (Figure 1). Notably, this strain is positive for 14-day arylsulfatase activity, urease, and tween 80 hydrolysis, but negative for niacin, nitrate, β-glucosidase, and catalase activity. A semiquantitative catalase test only resulted in a 3 mm column of effervescence above the medium surface. In addition to Middlebrook 7H10 agar and LJ medium, growth was observed on Löwenstein–Jensen with 5% sodium chloride medium, but not on MacConkey without crystal violet. *Mycobacterium salfingeri* sp. nov grew in the presence of mannitol but not with citrate or inositol. The capability for iron uptake was also measured and cells were negative for tellurite reduction. This strain was naturally resistant to doxycycline and tobramycin, and was intermediately susceptible to imipenem. At the genome level, the overall gene content, protein identities, and ANI scores were very dissimilar to any other NTM in our dataset, with only 41% of genes shared with its closest relative *M. celeriflavum*. *Mycobacterium salfingeri* sp. nov also harbors a 191,242 base pair plasmid that is only marginally similar to plasmid sequences available in public databases. The type strain is 20-157661T and was deposited at the Deutsche Sammlung von Mikroorganismen und Zellkulturen (DSMZ) and the Belgium Coordinated Collection of Microorganisms (BCCM) as DSM 113368T and ITM 501207T, respectively. Complete genome assembly and gene annotations have been deposited at the NCBI are accessible under the NCBI BioProject PRJNA753109.

## Data availability statement

The datasets presented in this study can be found in online repositories. The names of the repository/repositories and accession number(s) can be found at: NCBI – PRJNA753109, CP081005, and CP081006.

## Author contributions

EM and PL performed the bioinformatics analysis. CS performed the long-read DNA extraction and Nanopore NGS sequencing. AR, DK, and AF did the culture based phenotypic characterization. KM, VE, PL, and TH provided oversight, designed the experiments and wrote the manuscript. All authors contributed to the article and approved the submitted version.

## Conflict of interest

The authors declare that the research was conducted in the absence of any commercial or financial relationships that could be construed as a potential conflict of interest.

## Publisher’s note

All claims expressed in this article are solely those of the authors and do not necessarily represent those of their affiliated organizations, or those of the publisher, the editors and the reviewers. Any product that may be evaluated in this article, or claim that may be made by its manufacturer, is not guaranteed or endorsed by the publisher.

## Footnotes

^1^ https://www.ezbiocloud.net/

^2^ https://github.com/tseemann/abricate
