# Supplementary figures and images for: Characterization of Mycobacterium salfingeri sp. nov.: A novel nontuberculous mycobacteria isolated from a human wound infection
Source: Front Microbiol. 2022 Oct 10;13:992610. doi: 10.3389/fmicb.2022.992610 (PMC9589434; doi:10.3389/fmicb.2022.992610)

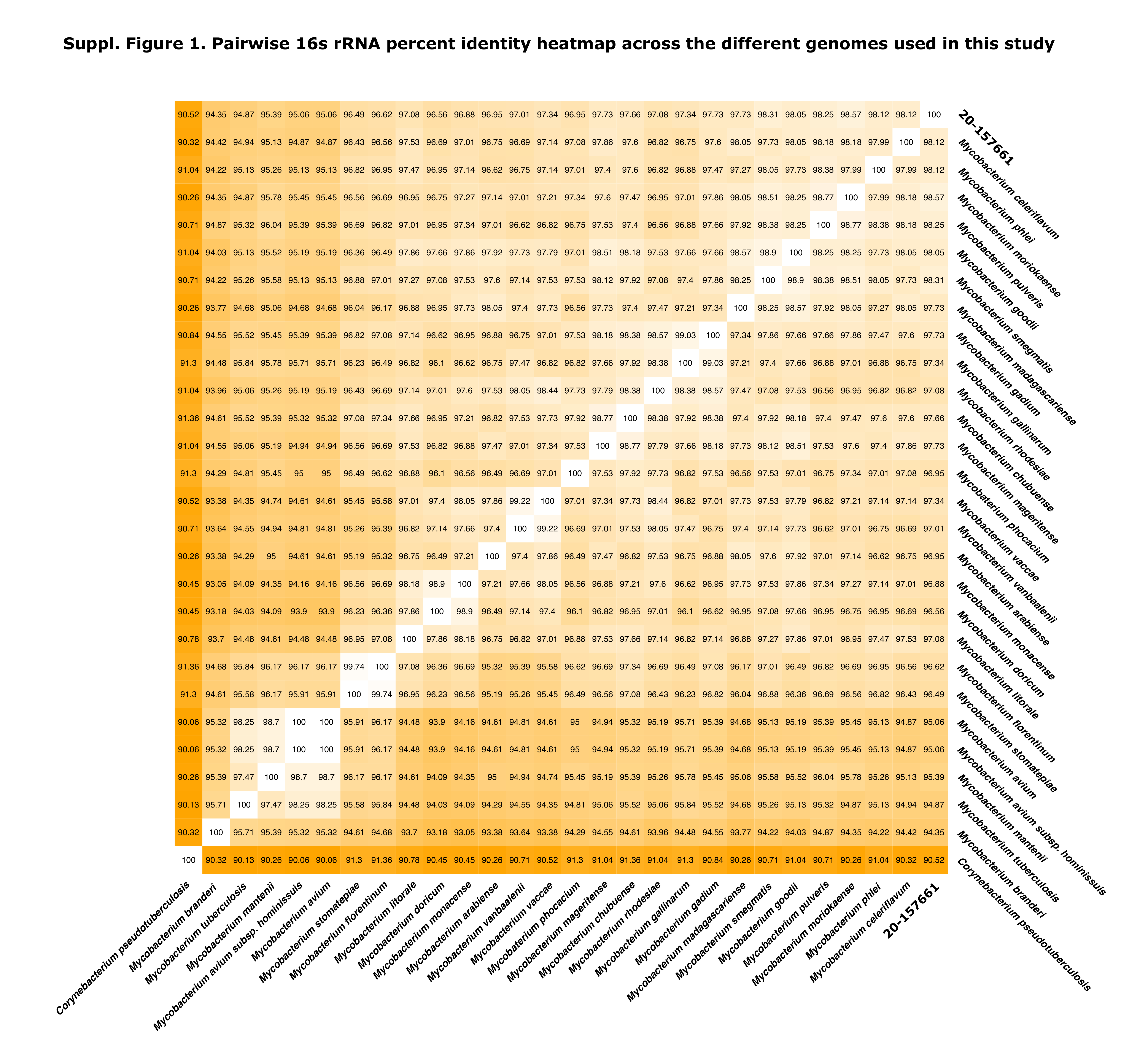

Supplement: Supplementary file 1 [file Image_1.jpeg]

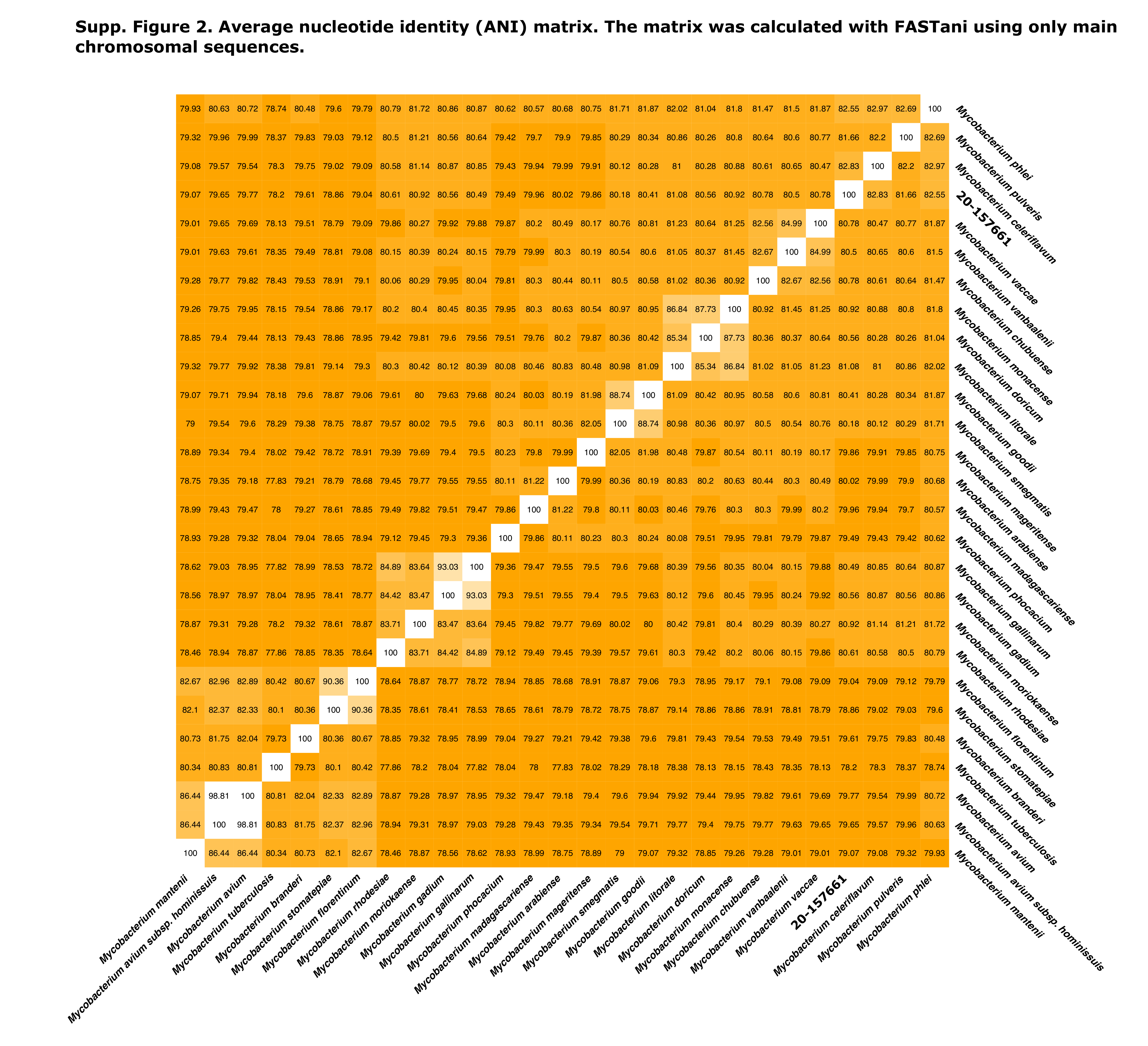

Supplement: Supplementary file 2 [file Image_2.jpeg]
